# The Precision-Recall Plot Is More Informative than the ROC Plot When Evaluating Binary Classifiers on Imbalanced Datasets

**DOI:** 10.1371/journal.pone.0118432

**Published:** 2015-03-04

**Authors:** Takaya Saito, Marc Rehmsmeier

**Affiliations:** Computational Biology Unit, Department of Informatics, University of Bergen, P. O. Box 7803, N-5020, Bergen, Norway; University of Louisville, UNITED STATES

## Abstract

Binary classifiers are routinely evaluated with performance measures such as sensitivity and specificity, and performance is frequently illustrated with Receiver Operating Characteristics (ROC) plots. Alternative measures such as positive predictive value (PPV) and the associated Precision/Recall (PRC) plots are used less frequently. Many bioinformatics studies develop and evaluate classifiers that are to be applied to strongly imbalanced datasets in which the number of negatives outweighs the number of positives significantly. While ROC plots are visually appealing and provide an overview of a classifier's performance across a wide range of specificities, one can ask whether ROC plots could be misleading when applied in imbalanced classification scenarios. We show here that the visual interpretability of ROC plots in the context of imbalanced datasets can be deceptive with respect to conclusions about the reliability of classification performance, owing to an intuitive but wrong interpretation of specificity. PRC plots, on the other hand, can provide the viewer with an accurate prediction of future classification performance due to the fact that they evaluate the fraction of true positives among positive predictions. Our findings have potential implications for the interpretation of a large number of studies that use ROC plots on imbalanced datasets.

## Introduction

Binary classifiers are statistical and computational models that divide a dataset into two groups, positives and negatives. They have been successfully applied to a wide range of biological and medical problems in recent years [1–3]. The evaluation of a classifier's prediction performance is of great importance in order to be able to judge its usefulness, also in comparison to competing methods. Commonly used measures of classifier performance in the phase of model construction are accuracy, error rate, and the Area under the Receiver Operating Characteristics (ROC) curve (AUC) [4]. Various additional measures are useful for the evaluation of the final model, and several plots provide visual representations, such as ROC and Precision-Recall (PRC) plots [5].

Class imbalance—a difference in the numbers of positive and negative instances, usually with the negatives outnumbering the positives—occurs in a wide range of scientific areas, including the life sciences, where unequal class distributions arise naturally [6–9]. The classification of imbalanced datasets is a relatively new challenge in the field of machine learning [5, 10]. While many solutions for binary classification on imbalanced data have been proposed [5, 11], they are mostly related to either data resampling [7, 12–14] or model-training [15–19]. Despite the development of state-of-the-art solutions for the building of classifiers with imbalanced data [5, 11, 20], selecting a suitable performance evaluation method is often underestimated.

It is important to recognize that the evaluation in the training phase is different from the evaluation of the final model. The first phase is to select the most effective and robust model during training. It usually divides a training dataset further into training and validation subsets, for example for cross-validation [21]. The second phase is to evaluate the final model after the training. Ideally, the test data of this phase reflects the class distributions of the original population even though such distributions are usually unknown. This article exclusively analyses the performance evaluation of the final model.

The rapid expansion in high-throughput biological experiments produces a number of large-sized datasets, and the majority of such datasets can be expected to be imbalanced [8, 22, 23]. Here, we review the theoretical background of commonly used evaluation measures, specifically ROC [24, 25], PRC [26], concentrated ROC (CROC) [27], and Cost Curves (CC) [28]. ROC is the most popular evaluation method for binary classifiers, but the interpretation of ROC curves requires a special caution when used with imbalanced datasets [29]. ROC alternatives, PRC, CROC, and CC, are less popular than ROC, but they are known to be robust even under imbalanced datasets [26–28]. In this study, we aim to clarify the difference between these measures from several different perspectives, addressing a computational-biology/life-sciences audience. To achieve this goal, we first introduce the basic single-threshold measures such as specificity and sensitivity, followed by the introduction of ROC and ROC alternative plots. We then discuss precision as an informative measure under imbalanced data as well as PRC that itself is based on precision. In a simulation study we analyse the behaviour and the utility of ROC, PRC, CROC, and CC when applied in the context of imbalanced datasets. The simulations use randomly generated samples with different performance levels. Subsequently, we show the results of a literature analysis that investigates what evaluation measures are used in real-world studies on imbalanced datasets. The literature analysis is based on two sets of PubMed search results. In addition, we re-analyse classifier performance from a previously published study, on a popular microRNA gene discovery algorithm called MiRFinder [30]. We also include a short review of available evaluation tools.

## Theoretical Background

Through the Theoretical Background section, we review the performance measures including basic measures from the confusion matrix and threshold-free measures such as ROC and PRC. We also include simple examples where necessary and a short introduction of tools. We use three distinct labels, ROC, PRC, and Tools, to organise the section. The first label, ROC, represents the theoretical background of basic measures, ROC, and ROC alternatives except PRC. The second label, PRC, represents the theoretical background of precision and PRC and comparisons between ROC and PRC. Finally, the third label, Tools, represents a short introduction of the tools for ROC, ROC alternatives, and PRC. We use these labels at the beginning of the sub-section titles to make the whole section easy to follow.

### ROC: Combinations of four outcomes in the confusion matrix form various evaluation measures

**Fig 1:**
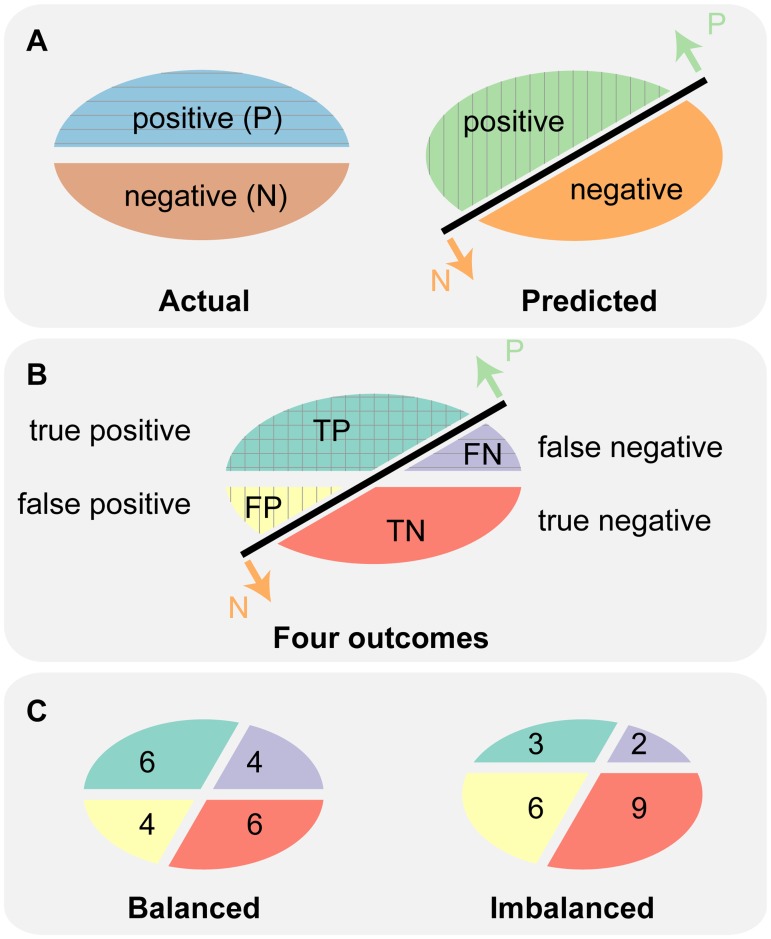
Actual and predicted labels generate four outcomes of the confusion matrix. (A) The left oval shows two actual labels: positives (P; blue; top half) and negatives (N; red; bottom half). The right oval shows two predicted labels: “predicted as positive” (light green; top left half) and “predicted as negative” (orange; bottom right half). A black line represents a classifier that separates the data into “predicted as positive” indicated by the upward arrow “P” and “predicted as negative” indicated by the downward arrow “N”. (B) Combining two actual and two predicted labels produces four outcomes: True positive (TP; green), False negative (FN; purple), False positive (FP; yellow), and True negative (TN; red). (C) Two ovals show examples of TPs, FPs, TNs, and FNs for balanced (left) and imbalanced (right) data. Both examples use 20 data instances including 10 positives and 10 negatives for the balanced, and 5 positives and 15 negatives for the imbalanced example.

In binary classification, data is divided into two different classes, positives (P) and negatives (N) (see Fig. 1A, left oval). The binary classifier then classifies all data instances as either positive or negative (see Fig. 1A, right oval). This classification produces four types of outcome—two types of correct (or true) classification, true positives (TP) and true negatives (TN), and two types of incorrect (or false) classification, false positives (FP) and false negatives (FN) (see Fig. 1B). A 2x2 table formulated with these four outcomes is called a confusion matrix. All the basic evaluation measures of binary classification are derived from the confusion matrix (see Table 1).

**Table 1:** Basic evaluation measures from the confusion matrix.

Measure	Formula
ACC	(TP + TN) / (TP + TN + FN + FP)
ERR	(FP + FN) / (TP + TN + FN + FP)
SN, TPR, REC	TP / (TP + FN)
SP	TN / (TN + FP)
FPR	FP / (TN + FP)
PREC, PPV	TP / (TP + FP)
MCC	(TP * TN—FP * FN) / ((TP + FP)(TP + FN)(TN + FP)(TN + FN))^1/2^
F_0.5_	1.5 * PREC * REC / (0.25 * PREC + REC)
F_1_	2 * PREC * REC / (PREC + REC)
F_2_	5 * PREC * REC / (4 * PREC + REC)

ACC: accuracy; ERR: error rate; SN: sensitivity; TPR: true positive rate; REC: recall; SP: specificity; FPR: false positive rate; PREC: precision; PPV: positive predictive value; MCC: Matthews correlation coefficient; F: F score; TP: true positives; TN: true negatives; FP: false positives; FN: false negatives

The most widely used basic measures of classifier performance are accuracy (ACC) and error rate (ERR) [5]. Sensitivity (SN) and specificity (SP) are also popular [31]. Sensitivity is equivalent to true positive rate (TPR) and recall (REC), and specificity is equivalent to 1—false positive rate (FPR). Another measure is precision (PREC), and PRC is based on it. Precision is also equivalent to positive predictive value (PPV).

Matthews correlation coefficient (MCC) [32] and F_β_ score [33] are also useful but less frequency used. MMC is a correlation coefficient calculated from all four values of the confusion matrix. The F_β_ score is a harmonic mean of recall and precision where β is commonly 0.5, 1, or 2.

All these measures have different advantages and disadvantages. Since they behave differently under balanced and imbalanced datasets, it is important to consider the class distribution of the data at hand or to be analysed in future applications and to select appropriate measures for meaningful performance evaluations.

### ROC: The ROC plot provides a model-wide evaluation of binary classifiers

Table 1 lists basic measures for the evaluation of classifier performance. All of these measures are single-threshold measures, that is, they are defined for individual score thresholds (cutoffs) of a classifier and cannot give an overview of the range of performance with varying thresholds. While any such threshold, which divides a dataset into positively and negatively predicted classes, can be reasonable in a particular application, it is not obvious how the right threshold value should be chosen. A powerful solution is to use threshold-free measures such as the ROC and PRC plots. These threshold-free measures require that classifiers produce some sort of scores from which the dataset can be divided into positively and negatively predicted classes, and not simply provide a static division. The majority of recent machine-learning libraries can produce discriminant values or posterior probabilities that can be used as scores [27, 34, 35], but not all classifiers provide such values.

The ROC plot shows the tradeoff between specificity and sensitivity [24]. It is model-wide because it shows pairs of specificity and sensitivity values calculated at all possible threshold scores. In ROC plots, classifiers with random performance show a straight diagonal line from (0, 0) to (1, 1) [24], and this line can be defined as the baseline of ROC. A ROC curve provides a single performance measure called the Area under the ROC curve (AUC) score. AUC is 0.5 for random and 1.0 for perfect classifiers [4]. AUC scores are convenient to compare the performances of multiple classifiers.

### ROC: The concentrated ROC (CROC) plot evaluates the early-retrieval performance of a classifier

**Fig 2:**
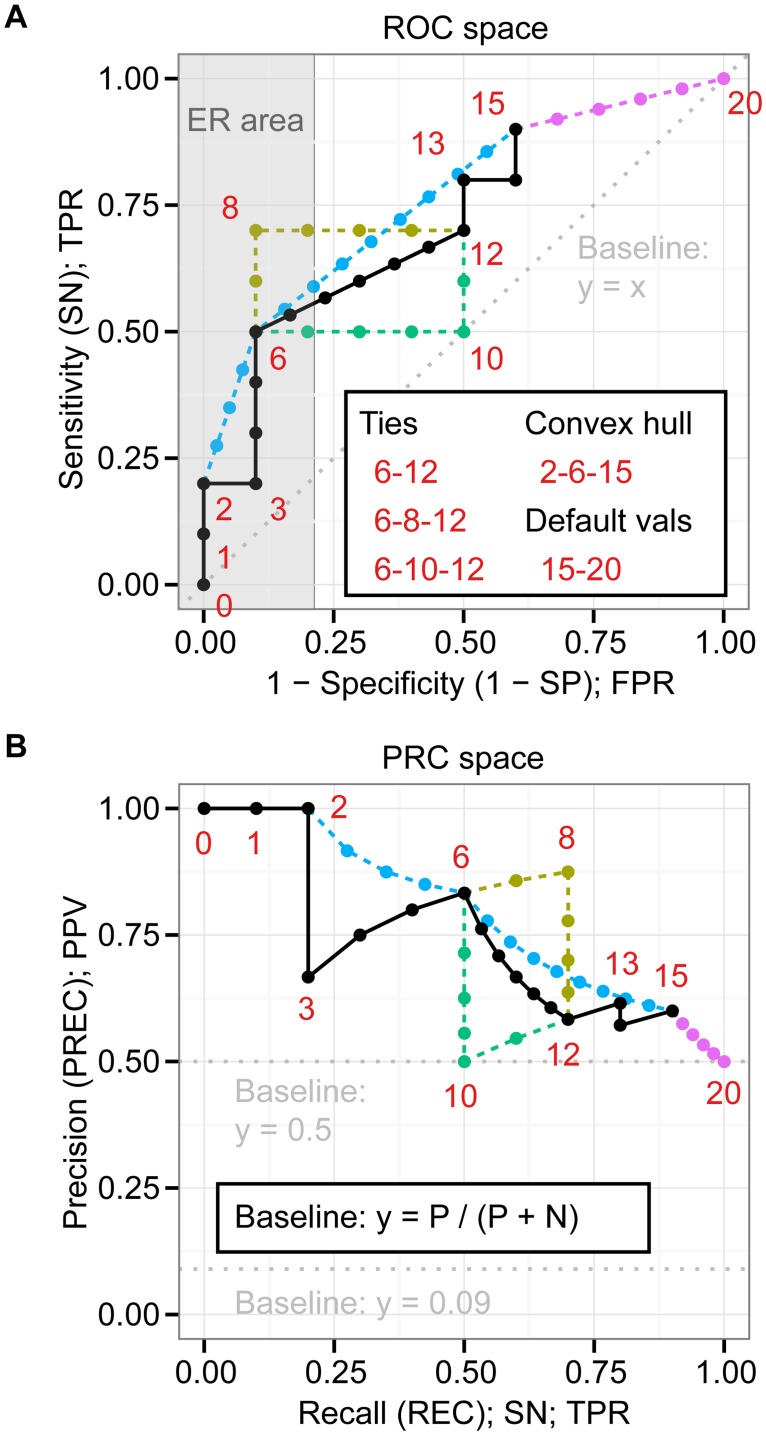
PRC curves have one-to-one relationships with ROC curves. (A) The ROC space contains one basic ROC curve and points (black) as well as four alternative curves and points; tied lower bound (green), tied upper bound (dark yellow), convex hull (light blue), and default values for missing prediction data (magenta). The numbers next to the ROC points indicate the ranks of the scores to calculate FPRs and TPRs from 10 positives and 10 negatives (See Table A in S1 File for the actual scores). (B) The PRC space contains the PR points corresponding to those in the ROC space.

The early retrieval (ER) area of a ROC plot (see the grey rectangle area in Fig. 2A) is useful for evaluating a fraction of the data with high-ranked instances [36, 37]. For example, if a classifier predicts a large part of the data as positive, it can be time-consuming and expensive to examine all the instances predicted as positive, especially when the dataset is large. Hence, it is practical to check the performance of the early retrievals, which only examines a limited number of top-scoring instances.

The concentrated ROC (CROC) plot facilitates the evaluation of early-retrieval performance [27]. A CROC plot is constructed with a magnifier function that transforms the FPRs on the x-axis. For instance, when using the exponential function with α = 7 (see Methods), this function transforms FPRs [0.0, 0.5, 1.0] into [0.0, 0.971, 1.0]. The region between 0 and 0.5 is expanded, whereas the region between 0.5 and 1.0 is shrunk. Similar to ROC plots, the area under the curve (AUC) of a CROC curve is likewise effective for classifier comparison [27]. While a simple, single-threshold measure such as ROC_50_, which sums up true positives (TPs) until the number of false positives (FPs) reaches 50 [38], can be useful in the evaluation of early-retrieval performance, the CROC plot provides a visual representation over the range of performances and with that a higher level of utility.

### ROC: The cost curve (CC) takes misclassification costs into account

The cost curve (CC) is an alternative to the ROC plot [12, 28]. Cost curves analyse classification performance by varying operating points [5]. Operating points are based on class probabilities and misclassification costs. The normalized expected cost or NE[C] represents the classification performance on the y-axis [28]. It is similar to the error rate, and therefore lower NE[C] values indicate better classifiers. The probability cost function (+) or PCF (+) represents the operating points on the x-axis [28]. PCF (+) is based on the probability of correctly classifying positives, and it is calculated by class probabilities and misclassification costs [5]. The actual calculations of PCF(+) and NE[C] are considerably more complex than the calculations involved in ROC and PRC plots (see Supplementary Methods in S1 File for the PCF(+) and NE[C] calculations).

### PRC: Precision is an intuitive measure when evaluating binary classifiers on imbalanced datasets

To investigate how the basic measures of classifier performance behave on balanced and imbalanced datasets, we created a simple example (see Fig. 1C). Both datasets have the same sample size. The numbers of true and false positive and negative predictions (TP, FP, TN, and FN) are defined as illustrated in Fig. 1C. Table 2 lists the results for the basic measures, as derived from the two datasets. Only precision, MMC, and the three F_β_ scores vary between the two datasets, while the majority of measures stay unchanged (see columns Balanced and Imbalanced in Table 2). More importantly, these unchanged measures fail to capture the poor performance of the classifier for the imbalanced sample. For example, accuracy (ACC) indicates that the performance of the classifier is fine for both samples (0.6). However, precision (PREC/PPV) indicates that the performance of the classifier is fine on the balanced (0.6) but relatively poor on the imbalanced dataset (0.33). Hence, precision reveals differences in performance that go unnoticed when using accuracy.

**Table 2:** Example of basic evaluation measures on a balanced and on an imbalanced dataset.

Measure	Balanced	Imbalanced
ACC	0.6	0.6
ERR	0.4	0.4
SN (TPR, REC)	0.6	0.6
SP	0.6	0.6
FPR	0.4	0.4
PREC (PPV)	0.6	0.33
MCC	0.2	0.17
F_0.5_	0.6	0.37
F_1_	0.6	0.43
F_2_	0.6	0.52

For the numbers of true and false positives and negatives in the two datasets, see Fig. 1C.

While MMC and the three F_β_ scores also vary between the two datasets, precision is easier to interpret. For instance, a precision of 0.33 can immediately be understood as 33% correct predictions among the positive predictions. This understanding directly translates to the application of the classifier to large datasets in which an estimate of the number of correct classifications among the positively classified instances (the "predictions") is of great importance. Precision is a direct and intuitive measure of this aspect of performance.

### PRC: The PRC plot shows the relationship between precision and sensitivity, and its baseline moves with class distribution

The precision-recall (PRC) plot shows precision values for corresponding sensitivity (recall) values. Similar to the ROC plot, the PRC plot provides a model-wide evaluation. The AUC score of PRC, denoted as AUC (PRC), is likewise effective in multiple-classifier comparisons [26].

While the baseline is fixed with ROC, the baseline of PRC is determined by the ratio of positives (P) and negatives (N) as y = P / (P + N). For instance, we have y = 0.5 for a balanced class distribution, but y = 0.09 for an imbalanced class distribution in which the ratio of P:N is 1:10 (see Fig. 2B). Because of this moving baseline, AUC (PRC) also changes with the P:N ratio. For instance, the AUC (PRC) of random classifiers is 0.5 only for balanced class distributions, whereas it is P / (P + N) for the general case, including balanced and imbalanced distributions. In fact, AUC (PRC) is identical to the y-position of the PRC baseline.

### PRC: PRC and ROC curves require different treatments when interpolating between points

A PRC curve has a one-to-one relationship with a corresponding ROC curve [26], that is, each point in any of the two curves uniquely determines a corresponding point in the other curve. Nonetheless, care must be taken when interpolations between points are performed, since the interpolation methods for PRC and ROC curves differ—ROC analysis uses linear and PRC analysis uses non-linear interpolation. Interpolation between two points A and B in PRC space can be represented as a function y = (TP_A_ + x) / {TP_A_ + x + FP_A_ + ((FP_B_ - FP_A_) * x) / (TP_B_ - TP_A_)} where x can be any value between TP_A_ and TP_B_ [26].

Three practical examples of ROC characteristics that associate with interpolation are ROC convex hull [39], the treatment of ties, and default values for missing scores. To explore these characteristics, we studied an example of 20 instances with an equal number of positives and negatives (see Fig. 2A; also see Table A in S1 File for scores and labels).

The ROC convex hull gives an estimate of the best possible performance of a classifier [39]. It is a combination of straight lines connecting only some of the points (0–2–6–13–15–20 in Fig. 2A), whereas the original ROC curve connects all the points with straight lines (all points from 0 to 20 in Fig. 2A). It is easy to see that the AUC of this ROC convex hull is better than that of the original ROC curve since some points are skipped for the ROC convex hull.

Classifiers sometimes produce ties (equal scores) for parts of the prediction (6–12 in Fig. 2A). Three obvious approaches to make a ROC curve from these ties are to use the upper bound with positive calculation first (6–8–12 in Fig. 2A), the lower bound with negative calculation first (6–10–12 in Fig. 2A), and the average (6–12 in Fig. 2A). ROC plotting tools normally use the average and lower bound methods [27, 40].

Classifiers sometimes fail to give scores to parts of the prediction. An example of such a case is the use of filtering before classification. Instances excluded by filtering likely have no scores assigned. In our example, the ROC plot shows a case in which the classifier gave scores for only 15 instances (0–15 in Fig. 2A) but not for the remaining five instances (16–20 in Fig. 2A). If the same default values are assigned to these five instances as a measure to compensate for missing scores, the ROC curve can linearly continue to the point (1, 1) (15–20 in Fig. 2A).

Although interpolation in PRC analysis requires more calculations than in ROC analysis, it is nonetheless critical to follow the correct procedure if misleading plots are to be avoided, especially when the distance of PRC points to be interpolated between is very large. The one-to-one relationship of individual points in our simple example can be seen on points 0–20 in Fig. 2A and B.

### Tools: A number of tools for making ROC and PRC plots are freely available

A number of tools for making ROC and PRC plots are freely available, but PRC functionality is generally deficient in comparison with ROC functionality. ROCR [40] is a popular R [41] package for drawing a variety of evaluation plots, including ROC, PRC, and CC. It lacks functionality for the calculation of non-linear PRC interpolations. AUCCalculator [26] is a Java application and provides accurate PRC and ROC interpolation. However, it lacks graph-plotting capability. CROC [27] is a Python package for CROC and ROC calculations. Several integrated machine learning and bioinformatics platforms such as WEKA [34] and Bioconductor [42, 43] also have basic functions or libraries for making ROC and PRC plots. Overall, a combination of AUCCalculator and any graph plotting program can be recommended for the creation of accurate PRC plots. ROCR can also be recommended, but only if interpolation between PRC points is not necessary.

## Material and Methods

### Basic evaluation measures

We calculated basic evaluation measures from a confusion matrix. The confusion matrix of binary classifiers has four outcomes, true positives (TP), true negatives (TN), false positives (FP), and false negatives (FN). The measures we discuss in this study are accuracy (ACC), error rate (ERR), sensitivity (SN), specificity (SP), true positive rate (TPR), recall (REC), false positive rate (FPR), precision (PREC), positive predictive value (PPV), Matthews correlation coefficient (MCC) [32], and F_β_ score [33] where β is 0.5, 1, or 2. Table 1 summarizes the formulae of these measures.

### Model-wide evaluation measures

The model-wide evaluation measures we analyse in our study are ROC, PRC, CROC, and CC. We used in-house Python and R scripts to calculate the values that are necessary to generate them. The scripts also include graph-plotting capability. The ROC plot has FPR or 1—specificity on the x-axis and TPR or sensitivity on the y-axis. The PRC plot has sensitivity/recall on the x-axis and precision/PPV on the y-axis. The CROC plot has transformed FPR on the x-axis and TPR on the y-axis. We used an exponential function, f(x) = (1 - exp(-αx))/(1 - exp(-α)) with α = 8 to transform the FPRs. The CC plot has the probability cost function (+) or PCF (+) on the x-axis and normalized expected cost or NE[C] on the y-axis [28]. PCF (+) is based on the probability of correctly classifying positives, whereas NE[C] represents the classification performance (see Supplementary Methods in S1 File for the PCF(+) and NE[C] calculations). We used AUCCalculator [26] and the CROC Python library [27] to calculate areas under the curve.

### Simulations with random sampling

**Fig 3:**
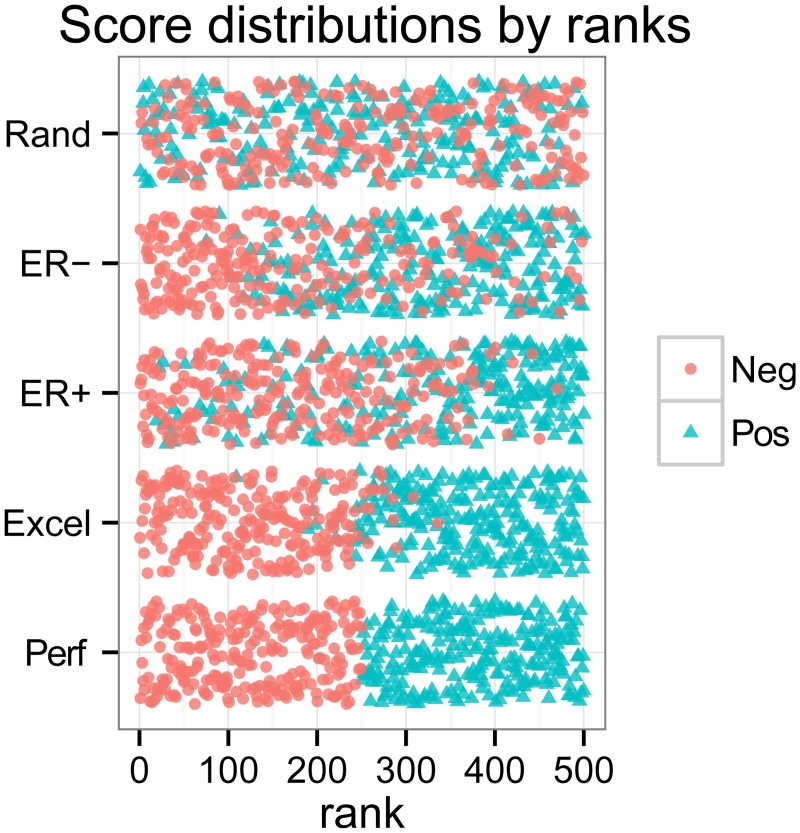
Combinations of positive and negative score distributions generate five different levels for the simulation analysis. We randomly sampled 250 negatives and 250 positives for Rand, ER-, ER+, Excel, and Perf, followed by converting the scores to the ranks from 1 to 500. Red circles represent 250 negatives, whereas green triangles represent 250 positives.

To analyse and compare the model-wide evaluation measures, we generated samples with five different levels of classifier performance by randomly drawing scores from score distributions for positives and negatives separately (Table 3). Instances with higher scores indicate that they are more likely labelled as positive. We sampled positives and negatives from a normal (N) or a Beta distribution to make four different levels, Random (Rand), Poor early retrieval (ER-), Good early retrieval (ER+), and Excellent (Excel). Instead of sampling from distributions, we used constant values 1 (positive) and 0 (negative) to make scores for Perfect (Perf). The scores of ER- and ER+ are based on similar score distributions. They differ in that ER+ tends to have more positives in higher (better) ranks, whereas ER- tends to have more positives in lower (worse) ranks. We stored the generated scores in arrays for sorting. We subsequently ranked them from the lowest to the highest scores. For tied scores, we assigned ranks in order of occurrence in the original array. Fig. 3 shows a visualisation of the score distributions for the five levels. In our simulation, we used 1000 positives and 1000 negatives for balanced datasets and 1000 positives and 10 000 negatives for imbalanced datasets. One round of simulation uses these samples to calculate all the necessary measures for ROC, PRC and the other plots. We then started again from the data sampling for another round. We iterated the whole process 1000 times. To plot the curves, we made 1000 bins for the x-axis and calculated the median of the corresponding values for the y-axis.

**Table 3:** Score distributions of positives and negatives for the performance simulations.

Level	Positives	Negatives
Random (Rand)	N(0, 1)	N(0, 1)
Poor early retrieval (ER-)	Beta(4, 1)	Beta(1, 1)
Good early retrieval (ER+)	Beta(1, 1)	Beta(1, 4)
Excellent (Excel)	N(3, 1)	N(0, 1)
Perfect (Perf)	1	0

N: normal distribution with mean and variance; Beta: Beta distribution with shape parameters. For performance level Perfect, fixed values 1 and 0 were used

### PubMed search

To investigate what evaluation measures are used for binary classifiers in life science studies, we performed two PubMed searches. In the first PubMed search, we aimed to find how popular ROC is in general and used the term “ROC OR (Receiver Operating Characteristics)”. From the results we collected the annual number of articles between 2002 and 2012. In the second PubMed search, we aimed to find genome-wide studies with Support Vector Machine classifiers and used the term “((Support Vector Machine) AND Genome-wide) NOT Association”. We used “Support Vector Machine” to find studies with binary classifiers and “Genome-wide” to find studies with imbalanced datasets. We also added “NOT Association” to exclude Genome-Wide Association Studies (GWAS) [44]. The search resulted in a list of 63 articles until May 2013 (Table B in S1 File). Three review articles and two articles that had no full-text access were excluded from further analysis.

### Literature analysis on the second PubMed search

We manually analysed the 58 articles retrieved by the second search and categorized them according to three main and 13 sub-categories (Tables C and D in S1 File). The three main categories are Type of SVM, Data type, and Evaluation method. We used the Type of SVM category to identify whether the SVM classifier is a binary classier. It contains two sub categories, BS (binary SVM) and OS (other SVM) (Table C in S1 File). We used the Data type category to identify whether the data set used for performance evaluation is imbalanced. It contains five sub categories, IB1 (strongly imbalanced), IB2 (imbalanced), SS (small sample size), BD (balanced data), and OD (other types of data) (Table C in S1 File). We used the Evaluation method category to identify what methods are used to evaluate the classification models. It contains five sub categories, ROC, STM1 (single-threshold measure only, group 1), PRC, pROC (partial ROC), STM2 (single-threshold measure only, group 2), and OE (other evaluation methods) (Table C in S1 File). We selected the sub-categories BS, IB1, IB2, SS, ROC, and PRC and calculated the proportions of articles for each sub-category against the total number of articles. Furthermore, we filtered the articles with the filter "BS and (IB1 or IB2) and not SS". The resulting 33 articles represent binary SVM classification studies with large size imbalanced data sets.

### Re-analysis of the MiRFinder study with ROC and PRC

**Fig 4:**
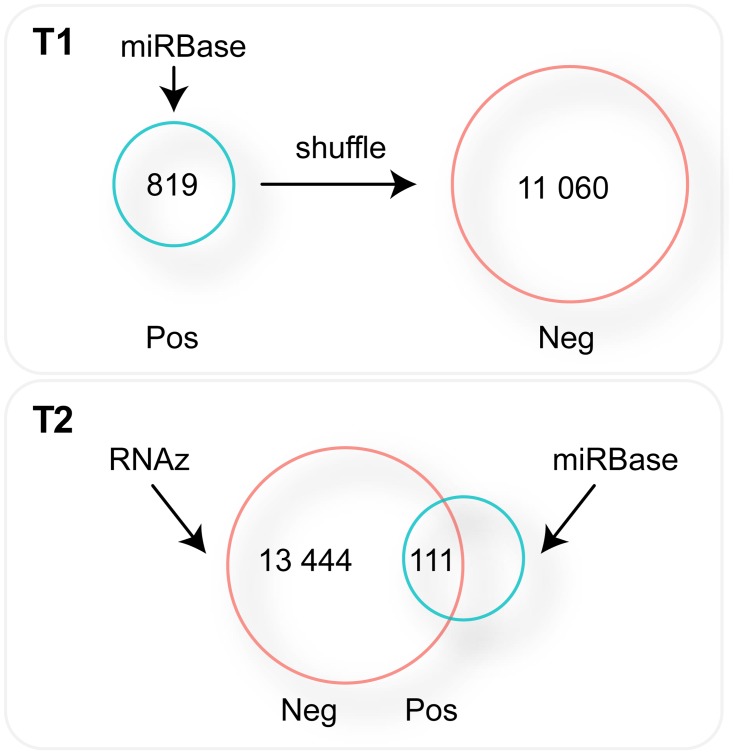
Simple scheme diagrams on the generation of datasets T1 and T2. T1 contains miRNA genes from miRBase as positives. Negatives were generated by randomly shuffling the nucleotides of the positives. For T2, the RNAz tool was used to generate miRNA gene candidates. Positives are candidate genes that overlap with the actual miRNA genes from miRBase.

We generated two test datasets for the re-analysis of the MiRFinder study and denoted them as T1 and T2 (Fig. 4). Dataset T1 uses actual miRNAs from several organisms for positives and pseudo-miRNAs generated by shuffling nucleotides of real miRNAs for negatives. Dataset T2 uses all functional RNA candidates generated by RNAz [45]. To extract the candidates, we used the whole *C*. *elegans* multiple alignment data with five worms (May 2008, ce6/WS190) from the University of California, Santa Cruz (USCS) Genome Bioinformatics site (http://genome.ucsc.edu). Positive candidates are those that overlap with miRBase [46] entries, and negatives are the remaining functional RNA candidates. T1 contains 819 positives and 11 060 negatives, and T2 contains 111 positives and 13 444 negatives. To calculate the scores of the miRNA discovery tools, we downloaded the source code of MiRFinder [30], miPred [47], RNAmicro [48], ProMir [49], and RNAfold [50] and installed them locally. We then calculated the scores of the tools on T1 and T2 (see Supplementary Methods in S1 File for more details on test data and score calculations).

## Results and Discussion

### Different perspectives on evaluation measures show that PRC is more informative than ROC with imbalanced datasets

Through the Results section, we aim to show how evaluation measures act under imbalanced datasets from several different perspectives. We use three distinct labels, Simulation, Literature analysis, and Re-analysis to organise the Results section. The first label, Simulation, represents a simulation analysis with randomly generated samples for ROC, CROC, CC, and PRC. The second label, Literature analysis, represents an analysis of the results from two sets of PubMed search to investigate the actual usage of evaluation measures in the life science literature. Finally, the third label, Re-analysis, represents a re-analysis of the MiRFinder study to reveal the difference between ROC and PRC in a real-world application. We use these labels at the beginning of the sub-section titles to make the whole Results section easy to follow.

### Simulation: The PRC plot is more informative than ROC, CROC, and CC plots when evaluating binary classifiers on imbalanced datasets

To investigate differences between ROC, CROC, CC, and PRC plots, we performed simulations with random sampling under balanced and imbalanced cases. To cover a wide range of practically relevant classifier behaviours, we studied five different performance levels—perfect, excellent, good early retrieval (ER+), poor early retrieval (ER-), and random—and generated scores by drawing randomly from different score distributions for positives and negatives separately (see Table 3). A balanced sample consisted of 1 000 positives and 1 000 negatives, and an imbalanced sample consisted of 1 000 positives and 10 000 negatives. Our observations about the four different types of plot are as follows.

**Fig 5:**
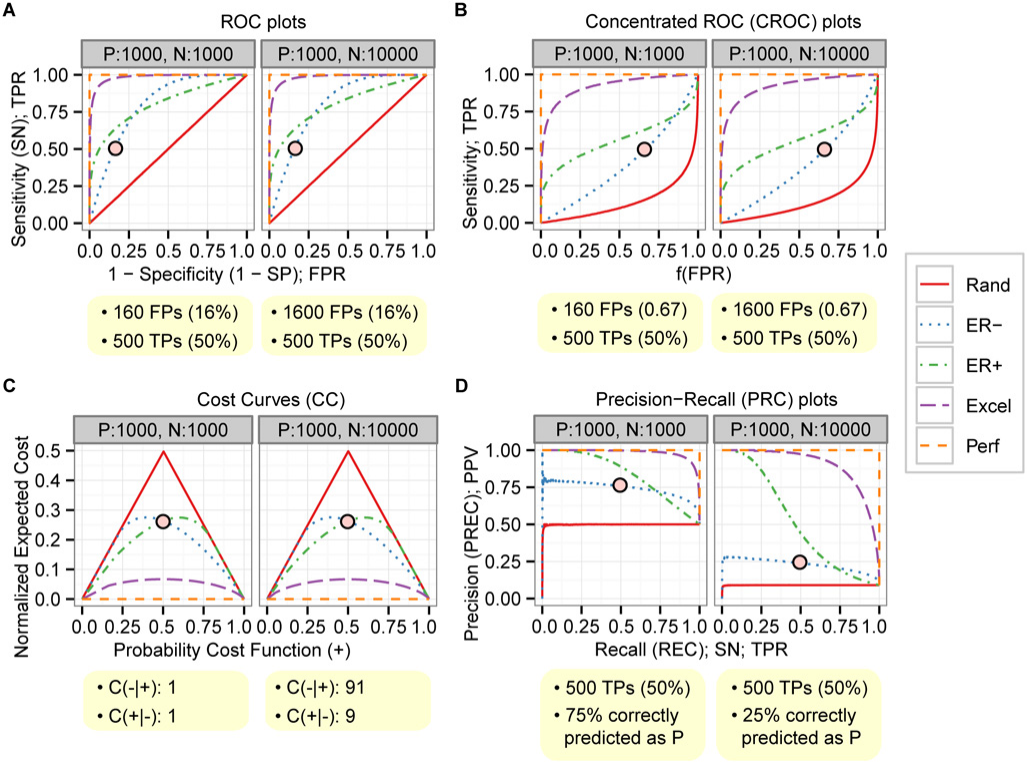
PRC is changed but the other plots are unchanged between balanced and imbalanced data. Each panel contains two plots with balanced (left) and imbalanced (right) for (A) ROC, (B) CROC with exponential function: f(x) = (1 - exp(-αx))/(1 - exp(-α)) where α = 7, (C) CC, and (D) PRC. Five curves represent five different performance levels: Random (Rand; red), Poor early retrieval (ER-; blue), Good early retrieval (ER+; green), Excellent (Excel; purple), and Perfect (Perf; orange).

**ROC plots.** The ROC plots are unchanged between balanced and imbalanced datasets (Fig. 5A), and all AUC (ROC) scores are unhanged accordingly (Table E in S1 File). Two points of ER- (red dots with black circle in Fig. 5A) are a good example to explain the difference of interpretations of the curves between balanced and imbalanced. The point for the balanced case represents 160 FPs and 500 TPs. ER- is likely considered a good classifier if this point is used for a performance evaluation. In contrast, the same point for the imbalanced case represents 1 600 FPs and 500 TPs, and the performance of the classifier is likely considered poor in this case. The ROC curves fail to explicitly show this performance difference. Moreover, it is also a good example to explain a potential mismatch between ROC curves in the early retrieval area and AUC (ROC). ER+ is clearly better than ER- in the early retrieval area, but AUC (ROC) scores are the same or 0.8 for both ER- and ER+ (Table E in S1 File). Therefore, AUC (ROC) is inadequate to evaluate the early retrieval performance in this case. Another potential problem is that AUC (ROC) can be inaccurate for fair comparisons when two ROC curves are crossing each other. The results of the simulations suggest that the interpretation of the ROC plot requires a special caution when the data are imbalanced and the early retrieval area needs to be checked.

**Concentrated ROC (CROC) plots.** Like the ROC plots, the CROC plots (Fig. 5B) are unchanged between balanced and imbalanced datasets. Accordingly, all AUC (CROC) scores are also unchanged (Table E in S1 File). Two points of ER- (red dots with black circle in Fig. 5B) represent a TPR of 0.5 and f(FPR) of 0.67. Since FPR is 0.16 when the f(FPR) is approximately 0.67, the points represent 500 TPs in both cases but 160 FPs in the balanced and 1 600 FPs in the imbalanced case. Similar to ROC, the CROC curves fail to explicitly show this performance difference. Nonetheless, the difference of the performances in the early retrieval area is clear because the area is widely expanded, which is the main advantage of CROC over ROC. Therefore, CROC can be useful when comparing the performance of classifiers in the early retrieval area. Nevertheless, CROC has the same issues as ROC in terms of the interpretation of the curves, especially when the dataset is imbalanced. Moreover, optimized parameters for magnifier functions, such as α, are usually unknown and difficult to decide, especially when multiple CROC curves cross each other.

**Cost curves (CC).** The CC plots are also unchanged between balanced and imbalanced datasets (Fig. 5C). CC is considerably different from the other ROC variants in terms of the interpretation of the plot. It shows the classification performances for different PCF (+) values that are based on misclassification costs and class probabilities. C(-|+) represents the cost of misclassifying positives as negatives, and C(+|-) represents the cost of misclassifying negatives as positives. p(+) and p(-) represent class probabilities for positives and negatives, respectively. Misclassification costs are often unknown but can for example be estimated from the class distributions. For instance, misclassification costs can be C(-|+) = 1 and C(+|-) = 1 for a balanced, and C(-|+) = 91 and C(+|-) = 9 for an imbalanced dataset. This would mean that the misclassification of positives as negatives is considerably more expensive than the misclassification of negatives as positives. To get the PCF(+) value of 0.5 (red dots with black circle in Fig. 5C), the corresponding class probabilities are p(+) = 0.5 and p(-) = 0.5 for the balanced dataset and p(+) = 0.09 and p(-) = 0.91 for the imbalanced dataset. Once the PCF (+) value of interest is determined, it is easy to compare the performances of multiple classifiers. Cost curves are useful when the testing of various misclassification costs and class probabilities is required, but a good understanding of PCF(+) and NE[C] is mandatory.

**Precision-Recall (PRC) plots.** In contrast to the ROC, CROC, and CC plots, the PRC plots are changed between balanced and imbalanced dataset (Fig. 5D). Accordingly, AUC (PRC) scores are also changed (Table E in S1 File). Two points of ER- (red dots with black circle in Fig. 5D) indicate that 75% and 25% are correct positive predictions in the balanced and in the imbalanced case, respectively, and these correct positive predictions are 50% of all positives. Hence, PRC correctly shows that the performance of ER- is good in the balanced but poor in the imbalanced case. The AUC (PRC) scores also support this (Table E in S1 File). Moreover, PRC shows that the performance of ER+ is better than ER- in both balanced and imbalanced cases. Again, AUC (PRC) scores support this (Table E in S1 File). In summary, PRC is able to show performance differences between balanced and imbalanced datasets, and it can be useful in revealing the early-retrieval performance.

**Summary of the simulation.** The overall results of the simulations suggest that PRC is the most informative and powerful plot for imbalanced cases and is able to explicitly reveal differences in early-retrieval performance.

### Literature analysis: The majority of studies on binary classifiers in conjunction with imbalanced datasets use the ROC plot as their main performance evaluation method

**Fig 6:**
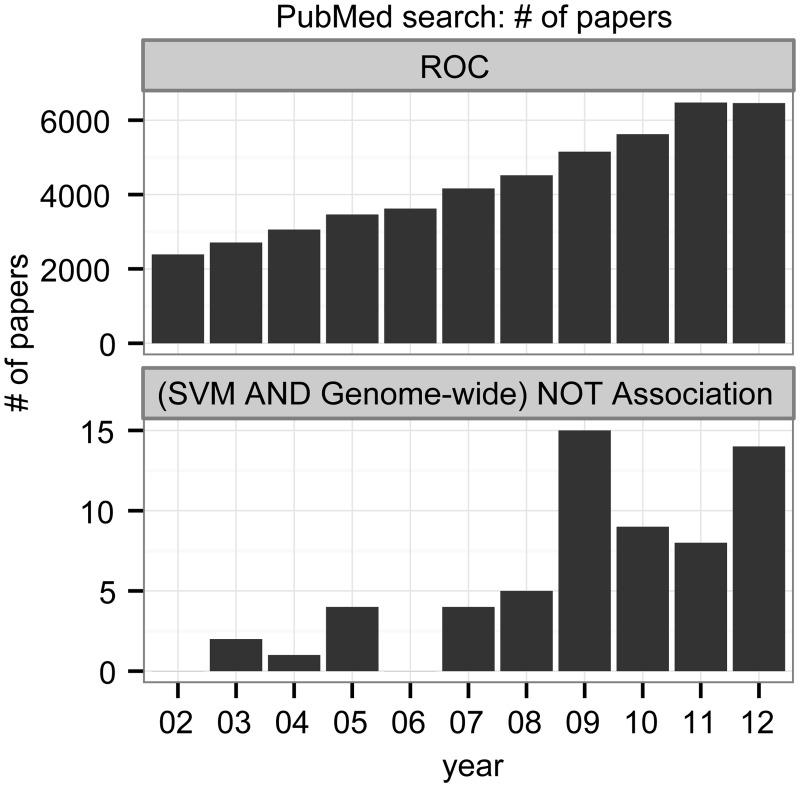
Two PubMed search results show the annual number of papers found between 2002 and 2012. The upper barplot shows the number of papers found by the term “ROC”, whereas the lower plot shows the number found by the term “((Support Vector Machine) AND Genome-wide) NOT Association”.

To assess to what degree our findings are relevant in practice, we analysed two sets of PubMed search results (see Methods). The goal of the first analysis was to determine quantitatively how popular ROC analysis is in general. The search result shows that ROC is indeed a popular method and that its popularity has been steadily increasing over the last decade (Fig. 6; upper panel).

The goal of the second analysis was to make a selection of binary-classification studies with imbalanced datasets for a further analysis. We used the PubMed term “((Support Vector Machine) AND Genome-wide) NOT Association” to find studies with imbalanced datasets that use support vector machines (SVMs) [51] for classification. The search resulted in 63 articles, of which 58 were research articles with full-text availability (Fig. 6; lower panel; see Table B in S1 File for a complete list of articles with references).

We categorized these 58 articles with respect to three categories: type of SVM, type of data, and evaluation method. The summarized result (Table 4) shows that the majority of the studies use SVM to build binary classifiers (Table 4; BS; 96.5%) and that more than half of the studies use imbalanced datasets (Table 4; B1 and IB2; 63.8%). As expected, ROC is the most popular method of performance evaluation (Table 4; ROC, All; 60.3%), and the proportion is even slightly increased after filtering by studies with binary classifiers with imbalanced datasets (Table 4; ROC, BS AND IB; 66.7%). This filtering also excludes studies with small sample size (Table 4; SS; 24.1%) since the approaches to solve imbalance problems with small sized data can be different from those of medium and large sized data [5, 52]. Only four papers use PRC (Table 4; PRC; 6.0%) as their evaluation method, whereas 22 papers use ROC (Table 4; ROC; 66.7%). Among them, three papers use both ROC and PRC. All of the remaining 10 papers use single threshold measures.

**Table 4:** Literature analysis summarized by three main categories and six subcategories.

Main^a^	Sub	Description	All^b^	BS AND IB^c^
SVM	BS	SVM binary classifiers	56 (95.6%)	33 (100%)
Data	IB1	Imbalanced (≥10-fold negatives)	28 (48.3%)	24 (72.7%)
	IB2	Imbalanced (2 to 9-fold negatives)	9 (15.5%)	9 (27.3%)
	SS	Small sample size (≤200)	14 (24.1%)	-
Eval	ROC	ROC or AUC (ROC)	35 (60.3%)	22 (66.7%)
	PRC	PRC or AUC (PRC)	4 (6.9%)	4 (12.1%)

^a^SVM: type of SVM, Data: data type, Eval: evaluation method.

^b^The total number of articles is 58.

^c^Filtered by SVM binary (BS) AND Imbalanced (IB1 or IB2) AND NOT Small sample size (SS). The total number of these articles is 33.

The results of the literature analysis clearly indicate that ROC is the most widely used evaluation method with imbalanced data, suggesting that changing the main evaluation method from ROC to PRC may influence many studies.

### Re-analysis: A re-evaluation of a previously published study confirms the advantages of the PRC plot over the ROC plot

To estimate how strongly studies of binary classifiers applied to imbalanced datasets could be affected by a change of the main evaluation method from ROC to PRC, we selected a study from our list of 58 research articles with full-text availability.

While the 58 studies vary across a wide range of research fields, five studies are from the field of microRNA (miRNA) gene discovery (Table F in S1 File). miRNAs are a class of small RNAs that have important regulatory roles in plants and animals [53], and finding genome locations of miRNA genes is a popular but challenging field in bioinformatics [54]. We selected the MiRFinder study [30] for re-analysis with PRC for three reasons: it uses ROC in conjunction with imbalanced data, the test data is available, and the classifier can produce scores, which is necessary for being able to create ROC and PRC plots.

The original MiRFinder study evaluates seven additional tools (Table G in S1 File). A ROC curve is only presented for the MiRFinder classifier itself, whereas ROC points (single points in ROC space) are provided for the other seven tools. From the seven additional tools evaluated in the MiRFinder study, we selected for our analysis the three tools that can produce scores and for which source code was available, namely miPred [47], RNAmicro [48], and ProMir [49], and added RNAfold [50] as a fourth tool. RNAfold predicts RNA secondary structure by minimizing over thermodynamic free energy. It is not a miRNA-specific tool, but the majority of miRNA gene discovery tools, including the four tools selected for our re-analysis, strongly rely on minimum free energy (MFE) calculations. It is thus interesting to determine how much additional performance the more sophisticated tools provide when compared to a baseline of RNAfold MFE calculations.

Since it is interesting to test performances under a variety of conditions, we added an additional test set. This test set was generated from the *C*. *elegans* genome by using the method described in the RNAmicro study [48].

**Fig 7:**
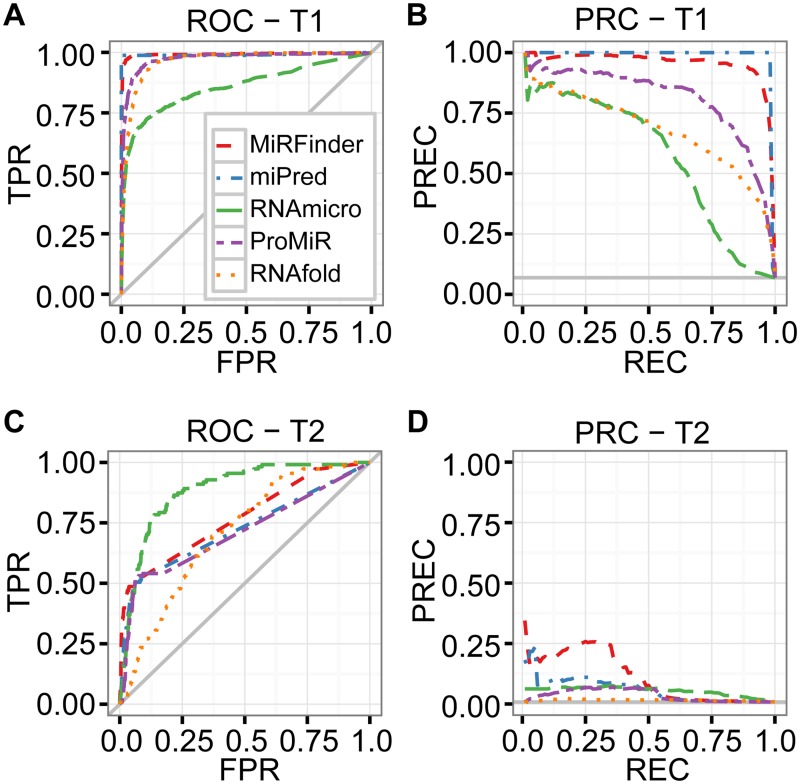
A re-analysis of the MiRFinder study reveals that PRC is stronger than ROC on imbalanced data. ROC and PRC plots show the performances of six different tools, MiRFinder (red), miPred (blue), RNAmicro (green), ProMiR (purple), and RNAfold (orange). A gray solid line represents a baseline. The re-analysis used two independent test sets, T1 and T2. The four plots are for (A) ROC on T1, (B) PRC on T1, (C) ROC on T2, and (D) PRC on T2.

In total, we evaluated five different tools on two independent test sets. We denote the test set from the MiRFinder study as T1 and the test set that we generated from the *C*. *elegans* genome as T2. The results of our evaluations are shown in Fig. 7 and Table 5 and are described and discussed in the following sub-sections.

**Table 5:** AUC scores of ROC and PRC for T1 and T2.

	T1		T2	
	ROC	PRC	ROC	PRC
MiRFinder	0.992*	0.945	0.772	0.106*
miPred	0.991	0.976*	0.707	0.024
RNAmicro	0.858	0.559	0.886*	0.054
ProMiR	0.974	0.801	0.711	0.035
RNAfold	0.964	0.670	0.706	0.015

Area under the curve (AUC) scores of ROC and PRC curves on datasets T1 and T2. The best AUC score in each column is marked with an asterisk (*).

### Re-analysis: PRC, but not ROC, reveals poor performance of some tools when tested on T1

**ROC on T1.** Fig. 7A indicates that all classifiers have a very good to excellent prediction performance. The top-performing classifiers, MiRFinder and miPred, have similar ROC curves, but miPred appears to perform better than MiRFinder in the early-retrieval area. The ROC plot does not immediately translate into an understanding of how reliable the predictions of the five tools will be and require some pondering about the practical meaning of the false positive rates shown. The AUC (ROC) scores (Table 5) indicate that MiRFinder is slightly better than miPred when studied over the whole range of FPRs, but this difference is too small to be of any practical relevance. The AUC (ROC) scores are in good agreement with the visual impression of the ROC plot, but likewise fail in terms of interpretability with respect to their practical meaning.

**PRC on T1.** Similar to the ROC plot in Fig. 7A, the PRC plot in Fig. 7B indicates that all classifiers have a very good to excellent prediction performance. However, we can see here that high recovery rates come with a deterioration of precision for some classifiers, especially for RNAmicro, but to a smaller extent also for RNAfold and ProMiR. We can also see that classifier performance is better resolved, allowing to spot differences more easily. Overall, the PRC plot allows for a quick and intuitive judgment of classifier performance, since it shows the tradeoff between the most relevant measures, precision and recall. The PRC plot in Fig. 7B also shows that all classifiers clearly distinguish themselves from a random classifier, indicated by the grey horizontal baseline. AUC (PRC) scores (Table 5) agree with the order of performance established in the PRC plot, but, being summaries of whole curves, cannot express the change of performance over the range of recall values.

### Re-analysis: PRC reveals very poor performance of all tools when tested on T2

**ROC on T2.** Fig. 7C shows a picture very different to Fig. 7A, which has to be attributed to differences in test data. RNAmicro is now clearly leading over a wide range of FPRs, although MiRFinder is stronger in the early-retrieval area. While in the mid-field of FPRs all methods show good performance, with RNAmicro showing very good performance, TPRs are low at small FPRs. In addition to the general difficulty with ROC plots of judging practical performance, Fig. 7C requires a decision about which areas of FPR are relevant and acceptable. A viewer might be tempted to be happy about the mid-FPR-field performance, not realizing that because of the strong imbalance of the data these FPRs could translate into large numbers of false-positive predictions. AUC (ROC) scores (Table 5) establish RNAmicro as the clear winner in this performance contest, naturally failing to express the change of performance over the range of FPR values, especially, in this case, in the early-retrieval area.

**PRC on T2.** Fig. 7D dramatically demonstrates that classifier performance deteriorates strongly under this test set. Over the whole range of recovery rates, all methods except MiRFinder have very low precision values, questioning their practical utility. MiRFinder performs relatively reasonably, with not extremely low precision at a not extremely low recovery rate, for example at 0.25/0.25. While the ROC plot in Fig. 7C makes an innocent impression, the PRC plot in Fig. 7D reveals the bitter truth. In the practically relevant measure of precision, all methods except MiRFinder have performances that are close to the performance of a random classifier, which is indicated by the grey horizontal line. Furthermore, the random-classifier baseline in Fig. 7D is lower than the one in Fig. 7B, expressing the stronger imbalance of the test data and the potential difficulty of constructing good classifiers. AUC (PRC) scores (Table 5) agree with the PRC plot in their ranking of the candidates, but, again naturally, cannot capture the variation of MiRFinder performance over the range of recovery rates.

### Re-analysis: PRC is more intuitive than other measures when tested on T1 and T2

**CROC and CC.** We also evaluated the five tools with CROC and CC, again on T1 and T2 (see Fig. A: A-D in S1 File). When compared to the ROC plots, the CROC plots (Fig. A: A-B in S1 File) show better resolution in the early-retrieval area, but are similarly unsuitable for a quick judgment of practical relevance. The interpretation is even more difficult, since the transformation function f has to be taken into account, although this could be remedied by annotating the x-axis with the original FPR values. As the ROC plots, the CROC plots do not show the full extent of performance deterioration under the T2 test set either. The same is true for the cost curves (Fig. A: C-D in S1 File), which are additionally unintuitive without a good understanding of NE[C] and PCF (+).

**Summary of the re-evaluation.** The results of our re-analysis clearly demonstrate the advantages of PRC against ROC. PRC plots show the practically relevant measures, precision and recall, of which precision is particularly important because it measures the fraction of correct predictions among the positive predictions. PRC plots express the susceptibility of classifiers to imbalanced datasets with clear visual cues. PRC plots are also useful for estimating the difficulty of creating a good classifier, since the position of the random baseline depends on the ratio of the numbers of positive and negative instances.

## Conclusion

ROC is a popular and strong measure to evaluate the performance of binary classifiers. However, it requires special caution when used with imbalanced datasets. CROC, CC, and PRC have been suggested as alternatives to ROC, but are less frequently used. In our comprehensive study, we show the differences between the various measures from several perspectives. Only PRC changes with the ratio of positives and negatives.

With the rapid expansion of high-throughput sequencing technology, the number of studies with machine leaning methods will likely increase. Our literature analysis suggests that the majority of such studies work with imbalanced datasets and use ROC as their main performance evaluation method. We have shown here that, unlike ROC plots, PRC plots express the susceptibility of classifiers to imbalanced datasets with clear visual cues and allow for an accurate and intuitive interpretation of practical classifier performance. The results of our study strongly recommend PRC plots as the most informative visual analysis tool.

## Supporting Information

S1 File Contains supplementary methods, one supplementary figure, seven supplementary tables, and supplementary references. Supplementary Methods. Cost curve calculations; preparations for the two independent test sets, T1 and T2; installation of four miRNA discovery tools and RNAfold; prediction scores of the five tools on T1 and T2. Figure A in S1 File. CROC and CC plots on test datasets T1 and T2. Table A in S1 File. Example of observed labels and predicted scores to make ROC and PRC curves to calculate interpolations. Table B in S1 File. List of 63 papers from PubMed search by “Support Vector Machine AND Genome-wide AND NOT Association”. Table C in S1 File. Descriptions of the three main and 13 sub categories. Table D in S1 File. Three main and 13 sub groups categorize the 58 research papers found by PubMed search. Table E in S1 File. AUC scores of ROC, PRC and CROC from the simulations with random sampling. Table F in S1 File. Five pre-miRNA studies selected from the literature analysis. Table G in S1 File. Seven tools used in the MiRFinder study for the comparisons. Supplementary References.
(DOCX)
