# Supplementary material for: The Precision-Recall Plot Is More Informative than the ROC Plot When Evaluating Binary Classifiers on Imbalanced Datasets
Source: PLoS One. 2015 Mar 4;10(3):e0118432. doi: 10.1371/journal.pone.0118432 (PMC4349800; doi:10.1371/journal.pone.0118432)
Supplement: S1 File — Supplementary Methods. Cost curve calculations; preparations for the two independent test sets, T1 and T2; installation of four miRNA discovery tools and RNAfold; prediction scores of the five tools on T1 and T2. Figure A in S1 File. CROC and CC plots on test datasets T1 and T2. Table A in S1 File. Example of observed labels and predicted scores to make ROC and PRC curves to calculate interpolations. Table B in S1 File. List of 63 papers from PubMed search by “Support Vector Machine AND Genome-wide AND NOT Association”. Table C in S1 File. Descriptions of the three main and 13 sub categories. Table D in S1 File. Three main and 13 sub groups categorize the 58 research papers found by PubMed search. Table E in S1 File. AUC scores of ROC, PRC and CROC from the simulations with random sampling. Table F in S1 File. Five pre-miRNA studies selected from the literature analysis. Table G in S1 File. Seven tools used in the MiRFinder study for the comparisons. Supplementary References. (DOCX) [file pone.0118432.s001.docx]

**Supplementary Information S1**

**The Precision-Recall plot is more informative and powerful than ROC to evaluate binary classifiers on imbalanced data.**

**Takaya Saito and Marc Rehmsmeier**

# Contents

## Supplementary Methods

| Cost curve calculations. |
| --- |
| Preparations for the two independent test sets, T1 and T2. |
| Installation of four miRNA discovery tools and RNAfold. |
| Prediction scores of the five tools on T1 and T2. |

## Supplementary Figure

| **Figure A** | CROC and CC plots on test datasets T1 and T2. |
| --- | --- |

## Supplementary Tables

| **Table A.** | Example of observed labels and predicted scores to make ROC and PRC curves to calculate interpolations. |
| --- | --- |
| **Table B.** | List of 63 papers from PubMed search by “Support Vector Machine AND Genome-wide AND NOT Association”. |
| **Table C.** | Descriptions of the three main and 13 sub categories. |
| **Table D.** | Three main and 13 sub groups categorize the 58 research papers found by PubMed search. |
| **Table E.** | AUC scores of ROC, PRC and CROC from the simulations with random sampling. |
| **Table F.** | Five pre-miRNA studies selected from the literature analysis. |
| **Table G.** | Seven tools used in the MiRFinder study for the comparisons. |

## Supplementary References

# Supplementary Methods

## Cost curve calculations.

NE[C] represents a normalized expected cost. Expected cost, E[C], is a value calculated from two error rates, (1 - TPR) and FPR with two weight values that are products of class distributions and misclassification costs.

| $E\left[ C \right]=\left( 1-TPR \right)*p\left( + \right)*C\left( - \vert+ \right)+ FPR*p\left( - \right)*C(+\vert-)$ | (1) |
| --- | --- |

Two weight values are p(+)C(-|+) and p(-)C(+|-) where p(+) and p(-) are class distributions and C(-|+) and C(+|-) are misclassification costs. p(+) and p(-) are respectively class distributions of positives and negatives. C(-|x) is a cost value of misclassifying positives as negatives, whereas C(+|-) is a cost value of misclassifying negatives as positives. The maximum value of E[C] is p(+)C(-|+) + p(-)C(+|-). NE[C] uses this maximum value for normalization.

| $NE\left[ C \right]=\frac{E[C]}{p\left( + \right)*C\left( - \vert+ \right)+ p\left( - \right)*C(+\vert-)}$ | (2) |
| --- | --- |

PCF(+) is a proportion of a weight value p(+)c(-|+) against the total of the weight values p(+)c(-|+) + p(+)c(-|+). The generalized version of PCF(+) is PCF(α).

| $PCF\left( \alpha\right)=\frac{p\left( \alpha\right)*C(\alpha\vert\bar{\alpha})}{p\left( + \right)*C\left( - \vert+ \right)+p\left( - \right)*C(+\vert-)}$ | (3) |
| --- | --- |

Hence,

| $PCF\left( + \right)=\frac{p\left( + \right)*C(+\vert-)}{p\left( + \right)*C\left( - \vert+ \right)+p\left( - \right)*C(+\vert-)}$ | (4) |
| --- | --- |

## Preparations for the two independent test sets, T1 and T2.

T1 is based on the independent data set used in MiRFinder [[1](#_ENREF_1)]. The original dataset contains 4473 positives and 11193 negatives. We filtered redundant entries out and excluded that use non-RNA letters, which resulted in 819 positives and 11060 negatives.

T2 is generated from the *C. elegans* genome by the method described in RNAmicro [[2](#_ENREF_2)]. First, we downloaded the *C. elegans* multiple alignment data with five worms (May 2008, ce6/WS190) from the University of California, Santa Cruz (USCS) Genome Bioinformatics site (http://genome.ucsc.edu) and *C. elegans* miRNA data (version 13.0, WS190) from miRBase [[3](#_ENREF_3)]. Second, we ran RNAz (version 2.1) [[4](#_ENREF_4)] downloaded from the RNAz web site (http://www.tbi.univie.ac.at/~wash/RNAz) with the window size 120 and the step size 50 on the multiple alignment data. Third, we filtered the RNAz output data and retained RNA structures predicted as ‘functional RNAs’, which resulted in 13555 candidate miRNA genes. Finally, we used real miRNA genes from miRBase and checked overlapped genome locations. We selected positive data if there are at least 20 overlapped nucleotides, which resulted in 111 positives and 13444 negatives.

## Installation of four miRNA discovery tools and RNAfold.

We downloaded the source code of five tools from the following locations:

MiRFinder (version 4.0) [[1](#_ENREF_1)] from http:// www.bioinformatics.org/mirfinder,

miPred [[5](#_ENREF_5)] from http://www.bioinf.seu.edu.cn/miRNA,

RNAmicro (version 1.1.3) [[2](#_ENREF_2)] from http://www.bioinf.uni-leipzig.de/~jana,

ProMiR (version 1.0) [[6](#_ENREF_6)] from http://bi.snu.ac.kr/ProMiR, and

RNAfold (version 1.85) from the ViennaRNA Package [[7](#_ENREF_7)] website (http://www.tbi.univie.ac.at/RNA/).

We complied them if necessary and locally installed them in our computer.

## Prediction scores of the five tools on T1 and T2.

Test files contain multiple alignments. Therefore, we changed the format of the test data depending on the capability of the tool for handling evolutionary information. For example, the T2 test data for miPred contain only *C. elegans* data, whereas the same T2 test data for RNAmicro contain *C. elegans* data as well as data from four other worms.

**MiRFinder**: The original version output only scores equal to or above 0.5, so we modified it to output any scores. The range of these scores is between 0.0 and 1.0. Since miRFinder gives no scores when the predicted secondary structure does not resemble miRNA precursors, we used the default value of -1.0 in such case.

**miPred**: miPred assigns three different classes to all the candidates: “real miRNA precursor”, “pseudo miRNA precursor”, and “no miRNA precursor hairpin”. The first two classes come with a confidence level in percentage. We used a value of the confidence level divided by 100 as the score for the first class. We used the same way as the first class, but then subtracted by 1 and the times -1 for the second class. For example, the score is 0.2 by (80 / 100 – 1) * -1 when the confidence level of the second class is 80%. Subsequently, the range of the scores for these two classes is between 0.0 and 1.0. We used the default values of -1.0 for the third class.

**RNAmicro**: RNAmicro produces scores between 0 and 1.0. We used three different window sizes, 70, 100, 130, and selected the best score. As like MiRFinder, RNAmicro gives no scores when the predicted secondary structure does not resemble miRNA precursors, we used the default value of -1.0 in such case.

**ProMiR**: ProMiR produces a pair of scores for 5’ to 3’ and 3’ to 5’ orientations. We selected the higher score of the two. All the scores are above 0. ProMiR also gives no scores when the predicted secondary structure does not resemble miRNA precursors, we used the default value of -1.0 in such case.

**RNAfold**: We used MFE * -1.0 as scores.

# Supplementary Figure

##
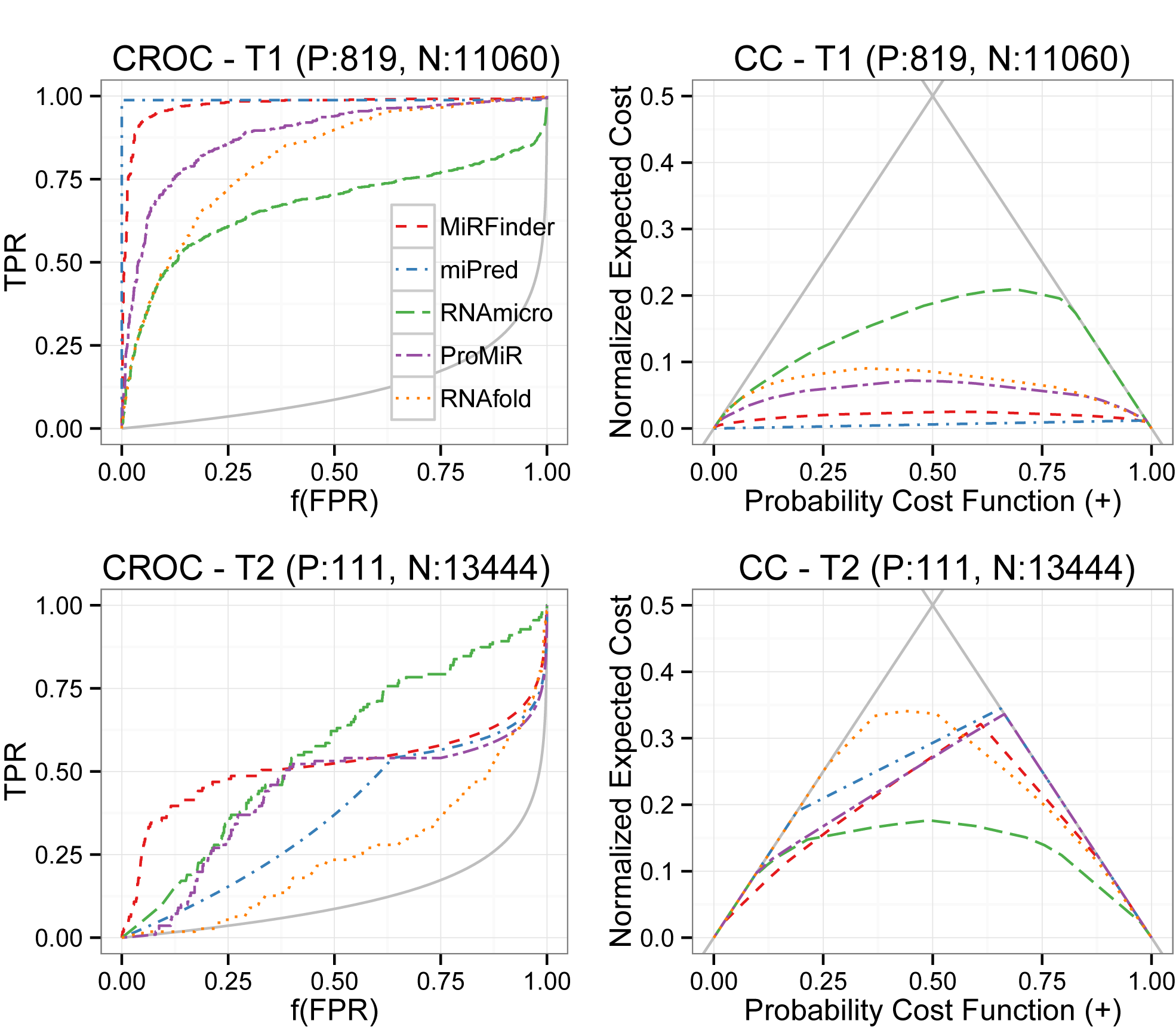


## Figure A - CROC and CC plots on test datasets T1 and T2.

Two CROC and two CC plots show the performance of six different tools, MiRFinder (red), miPred (blue), RNAmicro (green), ProMiR (purple), and RNAfold (orange). A gray solid line represents a baseline. Plots are for evaluating two independent test sets, T1 and T2, thus four plots as (A) CROC on T1, (B) CC on T1, (C) CROC on T2, and (D) CC on T2.

# Supplementary Tables

**Table A.** Example of observed labels and predicted scores to make ROC and PRC curves to calculate interpolations.

^1^Same point numbers used in Fig. 4 in the original paper. Points 1-15 of this table correspond to the black points in Fig. 4 in the original paper whereas points 16-20 correspond to the magenta points. ^2^Observed labels: positive (P) or negative (N). ^3^Preciction scores: Higher scores indicate that predicted labels are more likely “P”. ^4^Label counts for “P” to calculate TPs. 1 or 0 for non-tied scores whereas <1 for tied or interpolated scores. ^5^Label counts for “N” to calculate FPs.

| No. ^1^ | Obs^2^ | Score^3^ | L(P)^4^ | L(N)^5^ | TP | FP | TPR | FPR | PREC |
| --- | --- | --- | --- | --- | --- | --- | --- | --- | --- |
| 1 | P | 20 | 1 | 0 | 1.00 | 0.00 | 0.10 | 0.00 | 1.00 |
| 2 | P | 19 | 1 | 0 | 2.00 | 0.00 | 0.20 | 0.00 | 1.00 |
| 3 | N | 18 | 0 | 1 | 2.00 | 1.00 | 0.20 | 0.10 | 0.67 |
| 4 | P | 17 | 1 | 0 | 3.00 | 1.00 | 0.30 | 0.10 | 0.75 |
| 5 | P | 16 | 1 | 0 | 4.00 | 1.00 | 0.40 | 0.10 | 0.80 |
| 6 | P | 15 | 1 | 0 | 5.00 | 1.00 | 0.50 | 0.10 | 0.83 |
| 7 | P | 14 | 1/6*2 | 1/6*4 | 5.33 | 1.67 | 0.53 | 0.17 | 0.76 |
| 8 | P | 14 | 1/6*2 | 1/6*4 | 5.67 | 2.33 | 0.57 | 0.23 | 0.71 |
| 9 | N | 14 | 1/6*2 | 1/6*4 | 6.00 | 3.00 | 0.60 | 0.30 | 0.67 |
| 10 | N | 14 | 1/6*2 | 1/6*4 | 6.33 | 3.67 | 0.63 | 0.37 | 0.63 |
| 11 | N | 14 | 1/6*2 | 1/6*4 | 6.67 | 4.33 | 0.67 | 0.43 | 0.61 |
| 12 | N | 14 | 1/6*2 | 1/6*4 | 7.00 | 5.00 | 0.70 | 0.50 | 0.58 |
| 13 | P | 8 | 1 | 0 | 8.00 | 5.00 | 0.80 | 0.50 | 0.62 |
| 14 | N | 7 | 0 | 1 | 8.00 | 6.00 | 0.80 | 0.60 | 0.57 |
| 15 | P | 6 | 1 | 0 | 9.00 | 6.00 | 0.90 | 0.60 | 0.60 |
| 16 | N | 5 | 1/5*1 | 1/5*4 | 9.20 | 6.80 | 0.92 | 0.68 | 0.58 |
| 17 | P | 5 | 1/5*1 | 1/5*4 | 9.40 | 7.60 | 0.94 | 0.76 | 0.55 |
| 18 | N | 5 | 1/5*1 | 1/5*4 | 9.60 | 8.40 | 0.96 | 0.84 | 0.53 |
| 19 | N | 5 | 1/5*1 | 1/5*4 | 9.80 | 9.20 | 0.98 | 0.92 | 0.52 |
| 20 | N | 5 | 1/5*1 | 1/5*4 | 10.00 | 10.00 | 1.00 | 1.00 | 0.50 |

**Table B.** List of 63 papers from PubMed search by “Support Vector Machine AND Genome-wide AND NOT Association”.

| ID | Full Text | Research | Review | Ref |
| --- | --- | --- | --- | --- |
| 1 | O | O |  | [[8](#_ENREF_8)] |
| 2 | O | O |  | [[9](#_ENREF_9)] |
| 3 | O | O |  | [[10](#_ENREF_10)] |
| 4 | O | O |  | [[11](#_ENREF_11)] |
| 5 | O | O |  | [[12](#_ENREF_12)] |
| 6 | O | O |  | [[13](#_ENREF_13)] |
| 7 | O |  | O | [[14](#_ENREF_14)] |
| 8 | O | O |  | [[15](#_ENREF_15)] |
| 9 | O | O |  | [[16](#_ENREF_16)] |
| 10 | O |  | O | [[17](#_ENREF_17)] |
| 11 | O | O |  | [[18](#_ENREF_18)] |
| 12 | O | O |  | [[19](#_ENREF_19)] |
| 13 | O | O |  | [[20](#_ENREF_20)] |
| 14 | O | O |  | [[21](#_ENREF_21)] |
| 15 | O | O |  | [[22](#_ENREF_22)] |
| 16 |  | O |  | [[23](#_ENREF_23)] |
| 17 | O | O |  | [[24](#_ENREF_24)] |
| 18 | O | O |  | [[1](#_ENREF_1)] |
| 19 | O | O |  | [[25](#_ENREF_25)] |
| 20 | O | O |  | [[26](#_ENREF_26)] |
| 21 | O | O |  | [[27](#_ENREF_27)] |
| 22 | O | O |  | [[28](#_ENREF_28)] |
| 23 | O | O |  | [[29](#_ENREF_29)] |
| 24 | O |  | O | [[30](#_ENREF_30)] |
| 25 | O | O |  | [[31](#_ENREF_31)] |
| 26 | O | O |  | [[32](#_ENREF_32)] |
| 27 | O | O |  | [[33](#_ENREF_33)] |
| 28 | O | O |  | [[34](#_ENREF_34)] |
| 29 | O | O |  | [[35](#_ENREF_35)] |
| 30 | O | O |  | [[36](#_ENREF_36)] |
| 31 | O | O |  | [[37](#_ENREF_37)] |
| 32 | O | O |  | [[38](#_ENREF_38)] |
| 33 | O | O |  | [[39](#_ENREF_39)] |
| 34 | O | O |  | [[40](#_ENREF_40)] |
| 35 | O | O |  | [[41](#_ENREF_41)] |
| 36 | O | O |  | [[42](#_ENREF_42)] |
| 37 | O | O |  | [[43](#_ENREF_43)] |
| 38 | O | O |  | [[44](#_ENREF_44)] |
| 39 | O | O |  | [[45](#_ENREF_45)] |
| 40 | O | O |  | [[46](#_ENREF_46)] |
| 41 | O | O |  | [[47](#_ENREF_47)] |
| 42 | O | O |  | [[48](#_ENREF_48)] |
| 43 | O | O |  | [[49](#_ENREF_49)] |
| 44 | O | O |  | [[50](#_ENREF_50)] |
| 45 | O | O |  | [[51](#_ENREF_51)] |
| 46 |  | O |  | [[52](#_ENREF_52)] |
| 47 | O | O |  | [[53](#_ENREF_53)] |
| 48 | O | O |  | [[54](#_ENREF_54)] |
| 49 | O | O |  | [[55](#_ENREF_55)] |
| 50 | O | O |  | [[56](#_ENREF_56)] |
| 51 | O | O |  | [[57](#_ENREF_57)] |
| 52 | O | O |  | [[58](#_ENREF_58)] |
| 53 | O | O |  | [[59](#_ENREF_59)] |
| 54 | O | O |  | [[60](#_ENREF_60)] |
| 55 | O | O |  | [[61](#_ENREF_61)] |
| 56 | O | O |  | [[62](#_ENREF_62)] |
| 57 | O | O |  | [[63](#_ENREF_63)] |
| 58 | O | O |  | [[64](#_ENREF_64)] |
| 59 | O | O |  | [[65](#_ENREF_65)] |
| 60 | O | O |  | [[66](#_ENREF_66)] |
| 61 | O | O |  | [[67](#_ENREF_67)] |
| 62 | O | O |  | [[68](#_ENREF_68)] |
| 63 | O | O |  | [[69](#_ENREF_69)] |

**Table C.** Descriptions of the three main and 13 sub categories

| Main | Sub | Description |
| --- | --- | --- |
| SVM | BS | SVM binary classifiers. |
|  | OS | Other SVM models than regular binary classifiers, e.g. SVM multi-class classifiers. |
| Data | IB1 | Imbalanced data set with 10-fold or greater than 10-fold negatives to positives. |
|  | IB2 | Imbalanced data set with 2 to 9-fold negatives to positives. |
|  | SS | Small sample size (<200). |
|  | BD | Balanced data. |
|  | OD | Other data types than those described in the above, e.g. data with multiple classes and combination of several different types of data. |
| Eval | ROC | ROC curves and/or AUC (ROC) used for evaluation. |
|  | STM1 | No ROC curves used but at least one of the following single threshold measures used: ACC, ERR, SN, and SP. |
|  | PRC | PRC and/or AUC (PRC) used for evaluation. |
|  | pROC | Partial ROC, such as ROC_50,_ used for evaluation. |
|  | STM2 | No PRC used but at least one of the following single threshold measures used: PREC, MCC, and F_β_. |
|  | OE | Other evaluation methods than those described in the above or no evaluation methods used. |

**Table D.** Three main and 13 sub groups categorize the 58 research papers found by PubMed search.

^†^Same IDs used in Table B. ^1-3^See Table C for details.

| ID^†^ | SVM^1^ | | Data^2^ | | | | | Eval^3^ | | | | | |
| --- | --- | --- | --- | --- | --- | --- | --- | --- | --- | --- | --- | --- | --- |
|  | **BS** | **OS** | **IB1** | **IB2** | **SS** | **BD** | **OD** | **ROC** | **STM1** | **PRC** | **pROC** | **STM2** | **OE** |
| 1 | O |  |  | O |  |  |  | O |  | O | O |  |  |
| 2 | O |  | O |  |  |  |  |  |  | O |  |  |  |
| 3 | O |  | O |  |  | O |  | O |  |  |  | O |  |
| 4 | O |  |  |  |  |  | O |  |  |  |  |  | O |
| 5 | O |  |  | O |  |  |  | O |  |  |  |  |  |
| 6 | O |  |  |  | O |  |  |  | O |  |  |  |  |
| 8 | O |  |  |  | O |  |  | O |  |  |  |  |  |
| 9 | O |  |  |  | O |  |  | O |  |  |  |  |  |
| 11 | O |  | O |  |  |  |  | O |  | O |  |  |  |
| 12 | O |  | O |  |  |  |  |  | O |  |  |  |  |
| 13 | O |  | O |  |  |  |  | O |  |  |  | O |  |
| 14 | O |  |  |  | O | O |  |  | O |  |  |  |  |
| 15 | O |  | O |  |  | O |  |  | O |  |  |  |  |
| 17 | O |  | O |  |  | O |  | O |  |  |  | O |  |
| 18 | O |  |  | O |  | O |  | O |  |  |  |  |  |
| 19 | O |  |  | O |  |  |  |  | O |  |  |  |  |
| 20 | O |  | O |  |  | O |  |  | O |  |  | O |  |
| 21 | O |  | O |  |  | O |  | O |  |  |  |  |  |
| 22 | O |  | O |  |  |  |  | O |  |  |  |  |  |
| 23 | O |  | O |  |  |  |  | O |  |  |  | O |  |
| 25 | O |  |  |  | O |  |  |  | O |  |  |  |  |
| 26 | O |  | O |  |  |  |  | O |  |  |  |  |  |
| 27 |  | O | O |  |  |  |  |  |  |  |  |  | O |
| 28 | O |  |  |  |  |  | O | O |  |  |  |  |  |
| 29 | O |  | O |  |  |  |  | O |  |  | O |  |  |
| 30 | O |  |  |  |  |  | O | O |  |  |  | O |  |
| 31 | O |  |  | O |  | O |  |  | O |  |  |  |  |
| 32 | O |  |  | O |  |  |  | O |  |  |  | O |  |
| 33 | O |  |  |  | O |  |  | O |  |  |  |  |  |
| 34 | O |  |  |  | O |  |  |  | O |  |  |  |  |
| 35 | O |  |  | O |  |  |  | O |  |  |  |  |  |
| 36 | O |  | O |  |  | O |  | O |  |  |  | O |  |
| 37 | O |  | O |  |  |  |  |  | O |  |  | O |  |
| 38 | O |  | O |  |  | O |  | O |  |  |  |  |  |
| 39 | O |  |  |  |  |  | O |  | O |  |  |  |  |
| 40 | O |  |  |  |  | O |  | O |  |  |  |  |  |
| 41 | O |  |  |  |  |  | O | O |  |  | O |  |  |
| 42 | O |  | O |  |  |  |  |  | O |  |  | O |  |
| 43 | O |  |  |  | O |  |  |  | O |  |  |  |  |
| 44 | O |  | O |  |  |  |  | O |  |  | O | O |  |
| 45 | O |  | O |  |  |  |  |  | O |  |  |  |  |
| 47 | O |  | O |  |  |  |  | O |  | O |  |  |  |
| 48 | O |  |  |  | O |  |  | O |  |  |  |  |  |
| 49 | O |  | O |  |  |  |  | O |  |  |  |  |  |
| 50 | O |  |  |  |  |  | O | O |  |  |  | O |  |
| 51 | O |  |  | O |  |  |  | O |  |  |  | O |  |
| 52 | O |  | O |  |  |  |  | O |  |  |  |  |  |
| 53 | O |  |  |  |  |  | O | O |  |  |  | O |  |
| 54 | O |  |  |  | O |  |  |  | O |  |  |  |  |
| 55 |  | O | O |  |  |  |  |  |  |  |  |  | O |
| 56 | O |  |  |  | O |  |  | O |  |  |  | O |  |
| 57 | O |  |  |  |  | O |  | O |  |  | O |  |  |
| 58 | O |  | O |  |  |  |  |  | O |  |  |  |  |
| 59 | O |  |  |  | O |  |  |  | O |  |  |  |  |
| 60 | O |  | O |  |  |  |  | O |  |  |  |  |  |
| 61 | O |  |  | O |  |  |  |  | O |  |  | O |  |
| 62 | O |  | O |  | O | O |  |  | O |  |  |  |  |
| 63 | O |  | O |  | O |  |  | O |  |  |  |  |  |

**Table E.** AUC scores of ROC, PRC and CROC from the simulations with random sampling.

^1^B: balanced, IB: imbalanced.

| Level | B/IB^1^ | AUC (ROC) | AUC (PRC) | AUC (CROC) |
| --- | --- | --- | --- | --- |
| Perf | B | 1.0 | 1.0 | 1.0 |
| Excel | B | 0.98 | 0.98 | 0.92 |
| ER+ | B | 0.8 | 0.84 | 0.56 |
| ER- | B | 0.8 | 0.74 | 0.39 |
| Rand | B | 0.5 | 0.5 | 0.14 |
| Perf | IB | 1.0 | 1.0 | 1.0 |
| Excel | IB | 0.98 | 0.9 | 0.92 |
| ER+ | IB | 0.8 | 0.51 | 0.56 |
| ER- | IB | 0.8 | 0.23 | 0.39 |
| Rand | IB | 0.5 | 0.09 | 0.14 |

**Table F.** Five pre-miRNA studies selected from the literature analysis.

^†^Same IDs used in Table B.

| ID^†^ | Target species | For general use | Source code | Ref |
| --- | --- | --- | --- | --- |
| 15 | Fly | O |  | [[22](#_ENREF_22)] |
| 18 | Metazoan | O | O | [[1](#_ENREF_1)] |
| 27 | Medaka |  |  | [[33](#_ENREF_33)] |
| 49 | Metazoan | O |  | [[55](#_ENREF_55)] |
| 52 | Pig |  |  | [[58](#_ENREF_58)] |

**Table G.** Seven tools used in the MiRFinder study for the comparisons.

^1^Used for comparisons with the training dataset. ^2^Used for comparisons with the independent dataset ^3^Web interface is available as of September 2013. ^4^Source code is available as of September 2013. ^5^The tool can produce scores. ^6^Selected for re-reanalyzing the performance evaluation.

| Tool | Train^1^ | Ind^2^ | Web^3^ | Src^4^ | Score^5^ | Selected^6^ | Ref |
| --- | --- | --- | --- | --- | --- | --- | --- |
| miR-abela | O |  | O |  | O |  | [[70](#_ENREF_70)] |
| ProMir | O |  | O | O | O | O | [[6](#_ENREF_6)] |
| Triplet-SVM | O |  |  | O |  |  | [[71](#_ENREF_71)] |
| miRNA-SVM | O |  | O |  | O |  | [[72](#_ENREF_72)] |
| MiPred | O |  | O | O | O | O | [[5](#_ENREF_5)] |
| RNAmicro | O | O | O | O | O | O | [[2](#_ENREF_2)] |
| MiRScan | O | O |  |  |  |  | [[73](#_ENREF_73)] |

# Supplementary References

1. Huang, T.H., et al., *MiRFinder: an improved approach and software implementation for genome-wide fast microRNA precursor scans.* BMC Bioinformatics, 2007. **8**: p. 341.

2. Hertel, J. and P.F. Stadler, *Hairpins in a Haystack: recognizing microRNA precursors in comparative genomics data.* Bioinformatics, 2006. **22**(14): p. e197-202.

3. Kozomara, A. and S. Griffiths-Jones, *miRBase: integrating microRNA annotation and deep-sequencing data.* Nucleic Acids Res, 2011. **39**(Database issue): p. D152-7.

4. Gruber, A.R., et al., *RNAz 2.0: improved noncoding RNA detection.* Pac Symp Biocomput, 2010: p. 69-79.

5. Jiang, P., et al., *MiPred: classification of real and pseudo microRNA precursors using random forest prediction model with combined features.* Nucleic Acids Res, 2007. **35**(Web Server issue): p. W339-44.

6. Nam, J.W., et al., *Human microRNA prediction through a probabilistic co-learning model of sequence and structure.* Nucleic Acids Res, 2005. **33**(11): p. 3570-81.

7. Hofacker, I., et al., *Fast Folding and Comparison of RNA Secondary Structures.* Monatsh. Chem., 1994. **125**: p. 167-188.

8. Abraham, G., et al., *Performance and robustness of penalized and unpenalized methods for genetic prediction of complex human disease.* Genet Epidemiol, 2013. **37**(2): p. 184-95.

9. Bauer, T., R. Eils, and R. Konig, *RIP: the regulatory interaction predictor--a machine learning-based approach for predicting target genes of transcription factors.* Bioinformatics, 2011. **27**(16): p. 2239-47.

10. Bhasin, M., et al., *Prediction of methylated CpGs in DNA sequences using a support vector machine.* FEBS Lett, 2005. **579**(20): p. 4302-8.

11. Bolotin, E., et al., *Integrated approach for the identification of human hepatocyte nuclear factor 4alpha target genes using protein binding microarrays.* Hepatology, 2010. **51**(2): p. 642-53.

12. Chen, H.W., et al., *Predicting genome-wide redundancy using machine learning.* BMC Evol Biol, 2010. **10**: p. 357.

13. Chiang, C.Y., et al., *Cofactors required for TLR7- and TLR9-dependent innate immune responses.* Cell Host Microbe, 2012. **11**(3): p. 306-18.

14. Clarke, D., N. Bhardwaj, and M.B. Gerstein, *Novel insights through the integration of structural and functional genomics data with protein networks.* J Struct Biol, 2012. **179**(3): p. 320-6.

15. Daemen, A., et al., *Supervised classification of array CGH data with HMM-based feature selection.* Pac Symp Biocomput, 2009: p. 468-79.

16. Daemen, A., et al., *A kernel-based integration of genome-wide data for clinical decision support.* Genome Med, 2009. **1**(4): p. 39.

17. Dennis, J.L. and K.A. Oien, *Hunting the primary: novel strategies for defining the origin of tumours.* J Pathol, 2005. **205**(2): p. 236-47.

18. Eichner, J., et al., *Support vector machines-based identification of alternative splicing in Arabidopsis thaliana from whole-genome tiling arrays.* BMC Bioinformatics, 2011. **12**: p. 55.

19. Elstner, M., et al., *The mitochondrial proteome database: MitoP2.* Methods Enzymol, 2009. **457**: p. 3-20.

20. Fernandez, M. and D. Miranda-Saavedra, *Genome-wide enhancer prediction from epigenetic signatures using genetic algorithm-optimized support vector machines.* Nucleic Acids Res, 2012. **40**(10): p. e77.

21. Flanagan, J.M., et al., *DNA methylome of familial breast cancer identifies distinct profiles defined by mutation status.* Am J Hum Genet, 2010. **86**(3): p. 420-33.

22. Gu, J., et al., *Identifications of conserved 7-mers in 3'-UTRs and microRNAs in Drosophila.* BMC Bioinformatics, 2007. **8**: p. 432.

23. Gutlapalli, R.V., et al., *Genome wide search for identification of potential drug targets in Bacillus anthracis.* Int J Comput Biol Drug Des, 2012. **5**(2): p. 164-79.

24. Hegde, S.R., et al., *Understanding communication signals during mycobacterial latency through predicted genome-wide protein interactions and boolean modeling.* PLoS One, 2012. **7**(3): p. e33893.

25. Huang, W., N. Long, and H. Khatib, *Genome-wide identification and initial characterization of bovine long non-coding RNAs from EST data.* Anim Genet, 2012. **43**(6): p. 674-82.

26. Joung, J.G. and Z. Fei, *Computational identification of condition-specific miRNA targets based on gene expression profiles and sequence information.* BMC Bioinformatics, 2009. **10 Suppl 1**: p. S34.

27. Karklin, Y., R.F. Meraz, and S.R. Holbrook, *Classification of non-coding RNA using graph representations of secondary structure.* Pac Symp Biocomput, 2005: p. 4-15.

28. Kashima, H., et al., *Simultaneous inference of biological networks of multiple species from genome-wide data and evolutionary information: a semi-supervised approach.* Bioinformatics, 2009. **25**(22): p. 2962-8.

29. Kaundal, R., R. Saini, and P.X. Zhao, *Combining machine learning and homology-based approaches to accurately predict subcellular localization in Arabidopsis.* Plant Physiol, 2010. **154**(1): p. 36-54.

30. Keller, M.D. and S. Jyonouchi, *Chipping away at a mountain: Genomic studies in common variable immunodeficiency.* Autoimmun Rev, 2012.

31. Li, L., et al., *A robust hybrid between genetic algorithm and support vector machine for extracting an optimal feature gene subset.* Genomics, 2005. **85**(1): p. 16-23.

32. Li, L., et al., *Discovering cancer genes by integrating network and functional properties.* BMC Med Genomics, 2009. **2**: p. 61.

33. Li, S.C., et al., *Discovery and characterization of medaka miRNA genes by next generation sequencing platform.* BMC Genomics, 2010. **11 Suppl 4**: p. S8.

34. Liu, G., et al., *Sequence-dependent prediction of recombination hotspots in Saccharomyces cerevisiae.* J Theor Biol, 2012. **293**: p. 49-54.

35. Liu, H., et al., *Improving performance of mammalian microRNA target prediction.* BMC Bioinformatics, 2010. **11**: p. 476.

36. Lower, M. and G. Schneider, *Prediction of type III secretion signals in genomes of gram-negative bacteria.* PLoS One, 2009. **4**(6): p. e5917.

37. Lu, Y., et al., *Genome-wide computational identification of bicistronic mRNA in humans.* Amino Acids, 2013. **44**(2): p. 597-606.

38. Morita, K., et al., *Genome-wide searching with base-pairing kernel functions for noncoding RNAs: computational and expression analysis of snoRNA families in Caenorhabditis elegans.* Nucleic Acids Res, 2009. **37**(3): p. 999-1009.

39. Nam, H., et al., *Combining tissue transcriptomics and urine metabolomics for breast cancer biomarker identification.* Bioinformatics, 2009. **25**(23): p. 3151-7.

40. Nancarrow, D.J., et al., *Whole genome expression array profiling highlights differences in mucosal defense genes in Barrett's esophagus and esophageal adenocarcinoma.* PLoS One, 2011. **6**(7): p. e22513.

41. Okada, Y., K. Sato, and Y. Sakakibara, *Improvement of structure conservation index with centroid estimators.* Pac Symp Biocomput, 2010: p. 88-97.

42. Paladugu, S.R., et al., *Mining protein networks for synthetic genetic interactions.* BMC Bioinformatics, 2008. **9**: p. 426.

43. Puelma, T., R.A. Gutierrez, and A. Soto, *Discriminative local subspaces in gene expression data for effective gene function prediction.* Bioinformatics, 2012. **28**(17): p. 2256-64.

44. Qian, J., et al., *Prediction of regulatory networks: genome-wide identification of transcription factor targets from gene expression data.* Bioinformatics, 2003. **19**(15): p. 1917-26.

45. Rao, A., et al., *Motif discovery in tissue-specific regulatory sequences using directed information.* EURASIP J Bioinform Syst Biol, 2007: p. 13853.

46. Rawal, K. and R. Ramaswamy, *Genome-wide analysis of mobile genetic element insertion sites.* Nucleic Acids Res, 2011. **39**(16): p. 6864-78.

47. Reiche, K. and P.F. Stadler, *RNAstrand: reading direction of structured RNAs in multiple sequence alignments.* Algorithms Mol Biol, 2007. **2**: p. 6.

48. Rensing, S.A., et al., *Protein encoding genes in an ancient plant: analysis of codon usage, retained genes and splice sites in a moss, Physcomitrella patens.* BMC Genomics, 2005. **6**: p. 43.

49. Rha, S.Y., et al., *Prediction of high-risk patients by genome-wide copy number alterations from remaining cancer after neoadjuvant chemotherapy and surgery.* Int J Oncol, 2009. **34**(3): p. 837-46.

50. Sato, Y., A. Takaya, and T. Yamamoto, *Meta-analytic approach to the accurate prediction of secreted virulence effectors in gram-negative bacteria.* BMC Bioinformatics, 2011. **12**: p. 442.

51. Schweikert, G., et al., *mGene: accurate SVM-based gene finding with an application to nematode genomes.* Genome Res, 2009. **19**(11): p. 2133-43.

52. Shen, Y., Z. Liu, and J. Ott, *Support vector machines with L1 penalty for detecting gene-gene interactions.* Int J Data Min Bioinform, 2012. **6**(5): p. 463-70.

53. Sonnenburg, S., et al., *Accurate splice site prediction using support vector machines.* BMC Bioinformatics, 2007. **8 Suppl 10**: p. S7.

54. Tomas, G., et al., *A general method to derive robust organ-specific gene expression-based differentiation indices: application to thyroid cancer diagnostic.* Oncogene, 2012. **31**(41): p. 4490-8.

55. van der Burgt, A., et al., *In silico miRNA prediction in metazoan genomes: balancing between sensitivity and specificity.* BMC Genomics, 2009. **10**: p. 204.

56. Wang, C., et al., *Accurate prediction of the burial status of transmembrane residues of alpha-helix membrane protein by incorporating the structural and physicochemical features.* Amino Acids, 2011. **40**(3): p. 991-1002.

57. Wang, Y., et al., *High-accuracy prediction of bacterial type III secreted effectors based on position-specific amino acid composition profiles.* Bioinformatics, 2011. **27**(6): p. 777-84.

58. Wang, Z., et al., *The prediction of the porcine pre-microRNAs in genome-wide based on support vector machine (SVM) and homology searching.* BMC Genomics, 2012. **13**: p. 729.

59. Won, H.H., et al., *Cataloging coding sequence variations in human genome databases.* PLoS One, 2008. **3**(10): p. e3575.

60. Wu, B., et al., *Comparison of statistical methods for classification of ovarian cancer using mass spectrometry data.* Bioinformatics, 2003. **19**(13): p. 1636-43.

61. Xiao, X., et al., *Genome-wide identification of Polycomb target genes in human embryonic stem cells.* Gene, 2013.

62. Xu, H., I.R. Lemischka, and A. Ma'ayan, *SVM classifier to predict genes important for self-renewal and pluripotency of mouse embryonic stem cells.* BMC Syst Biol, 2010. **4**: p. 173.

63. Xu, X., Y. Ji, and G.D. Stormo, *Discovering cis-regulatory RNAs in Shewanella genomes by Support Vector Machines.* PLoS Comput Biol, 2009. **5**(4): p. e1000338.

64. Yellaboina, S., K. Goyal, and S.C. Mande, *Inferring genome-wide functional linkages in E. coli by combining improved genome context methods: comparison with high-throughput experimental data.* Genome Res, 2007. **17**(4): p. 527-35.

65. Zamanian, M., et al., *The repertoire of G protein-coupled receptors in the human parasite Schistosoma mansoni and the model organism Schmidtea mediterranea.* BMC Genomics, 2011. **12**: p. 596.

66. Zeng, J., et al., *Genome-wide polycomb target gene prediction in Drosophila melanogaster.* Nucleic Acids Res, 2012. **40**(13): p. 5848-63.

67. Zhang, X., et al., *The Trypanosoma brucei MitoCarta and its regulation and splicing pattern during development.* Nucleic Acids Res, 2010. **38**(21): p. 7378-87.

68. Zhang, Y., et al., *Network motif-based identification of transcription factor-target gene relationships by integrating multi-source biological data.* BMC Bioinformatics, 2008. **9**: p. 203.

69. Zinzen, R.P., et al., *Combinatorial binding predicts spatio-temporal cis-regulatory activity.* Nature, 2009. **462**(7269): p. 65-70.

70. Sewer, A., et al., *Identification of clustered microRNAs using an ab initio prediction method.* BMC Bioinformatics, 2005. **6**: p. 267.

71. Xue, C., et al., *Classification of real and pseudo microRNA precursors using local structure-sequence features and support vector machine.* BMC Bioinformatics, 2005. **6**: p. 310.

72. Helvik, S.A., O. Snove, Jr., and P. Saetrom, *Reliable prediction of Drosha processing sites improves microRNA gene prediction.* Bioinformatics, 2007. **23**(2): p. 142-9.

73. Lim, L.P., et al., *The microRNAs of Caenorhabditis elegans.* Genes Dev, 2003. **17**(8): p. 991-1008.
